# Salinity tolerance and geographical origin predict global alien amphipod invasions

**DOI:** 10.1098/rsbl.2020.0354

**Published:** 2020-09-02

**Authors:** Ross N. Cuthbert, Syrmalenia G. Kotronaki, Jaimie T. A. Dick, Elizabeta Briski

**Affiliations:** 1 GEOMAR, Helmholtz-Zentrum für Ozeanforschung Kiel, Kiel, Germany; 2 Institute for Global Food Security, School of Biological Sciences, Queen's University Belfast, Belfast, UK

**Keywords:** aquatic aliens, biodiversity, invasion success, Ponto-Caspian, predicting invaders, salinity regime

## Abstract

Invasive alien species are driving global biodiversity loss, compromising ecosystem function and service provision, and human, animal and plant health. Habitat characteristics and geographical origin may predict invasion success, and in aquatic environments could be mediated principally by salinity tolerance. Crustacean invaders are causing global problems and we urgently require better predictive power of their invasiveness. Here, we compiled global aquatic gammarid (Crustacea: Amphipoda: Gammaroidea) diversity and examined their salinity tolerances and regions of origin to test whether these factors predict invasion success. Across 918 aquatic species within this superfamily, relatively few gammarids (*n* = 27, 3%) were reported as aliens, despite extensive invasion opportunities and high numbers of published studies on amphipod invasions. However, reported alien species were disproportionately salt-tolerant (i.e. 32% of brackish-water species), with significantly lower proportions of aliens originating from freshwater and marine environments (both 1%). Alien gammarids also significantly disproportionally originated from the Ponto-Caspian (20% of these taxa) when compared with all ‘other' grouped regions (1%), and principally invaded Eurasian waters, with translocations of salt-tolerant taxa to freshwaters being pervasive. This suggests habitat characteristics, alongside regional contexts, help predict invasibility. In particular, broad environmental tolerances to harsh environments and associated evolutionary history probably promote success of aliens globally.

## 1. Introduction

The translocation of alien species to novel regions is one defining feature of anthropogenic global change [1], and this spread has increased in recent decades with no sign of saturation [2]. Globalization of trade and transport networks has intensified, with alien species transported via a range of human-mediated vectors which circumvent natural biogeographic barriers [1,3–5]. Invasive alien species are a leading cause of ongoing global biodiversity loss, causing substantial changes to food webs and ecosystem functioning [6], and aliens are driving extinctions from local to global scales [7,8]. However, the multi-stage process of biological invasion, including transport, introduction, establishment and spread, acts as an often unpredictable impediment to invasion success, with introduced taxa frequently failing to establish in novel habitats [9]. However, characteristics of individuals from alien populations, as well as those of origin environments, might mediate the success of alien species in new environments [10]. In particular, phenotypic plasticity and preadaptation to changeable environments are thought to assist alien species in withstanding the invasion process and establishing new and viable populations [11–13]. This conjecture, however, still lacks rigorous testing and thus our predictive power for new invasions remains low.

Shipping, aquaculture and canal construction have facilitated thousands of alien species becoming established in freshwater, brackish and marine ecosystems worldwide [14]. Salinity regime tolerance is thought to be a primary determinant of species distributions in aquatic environments [15], and species translocations among salinity regimes may exhibit unidirectional patterning. However, current invasion theories are underpinned by the concept that evolutionary experience can determine invasion success and impacts, or lack thereof [16]. Broadly speaking, evolutionary experience of physiologically harsh environments, such as transitional (or brackish) waters and their associated fluctuating environmental parameters (e.g. oxygen), might predispose species to invading other regions, including degraded and raised salinity freshwaters [17,18]. However, while colonization by alien taxa between specific regions has been examined (e.g. from Ponto-Caspian to North and Baltic Seas and Great Lakes-St Lawrence River) [12], whether invasion success is predicted by salinity tolerance and region of origin has not been explicitly tested with statistical rigour. In turn, this hampers predictions of aquatic species redistributions as globalization intensifies and the availability of non-native source pools increases [4,5].

Here, we thus determine, for a well-studied group that contributes to global invasions, (i) whether alien species with salinity tolerances are disproportionately predisposed to become alien, and (ii) whether the origin of alien species is disproportionately from suspected donor ‘hotspots', such as the Ponto-Caspian region. We consider the salinity tolerances and origins of gammarid crustaceans (Amphipoda: Gammaroidea). Globally, gammarids are a diverse and widespread group, with representatives across a range of aquatic habitats, and which have been intensively studied by invasion scientists, e.g. [19]. We thus compiled the total known aquatic biodiversity of gammarids, their salinity tolerances and geographical origins, enabling key salinity ‘donor' characteristics and also ‘donor–recipient' region linkages to be identified. Owing to the global notoriety of the Ponto-Caspian region as the origin of many invasive alien species [11], we hypothesized geographical biases towards this region characterized by its salt-tolerant species.

## 2. Material and methods

Total global biodiversity of fully aquatic gammarids was determined from the World Amphipod Database, reported in the World Register of Marine Species in February 2017. Each species was categorized against a number of key database descriptors. First, for all species captured, alien status was derived, i.e. whether the species is known to have translocated and established outside of its native range. Second, geographical origin and invaded region if applicable, as well as salinity categorizations for these areas (freshwater, less than 0.5 ppt; brackish, 0.5–30 ppt; marine, greater than 30 ppt) [20], were obtained for each species. For our analysis purposes, species which predominantly tolerated freshwaters were categorized as ‘freshwater', those which are well-known to tolerate freshwater up to brackish environments were ‘salt-tolerant', and those which predominantly tolerated fully marine conditions were deemed ‘marine'. Regional categorizations were made following Casties *et al*. [12]: northeast Atlantic, northwest Atlantic, southeast Atlantic, southwest Atlantic, northeast Pacific, northwest Pacific, southeast Pacific, southwest Pacific, North Sea, Baltic Sea, the Great Lakes–St Lawrence River region, Mediterranean Sea, Eurasia (inland freshwaters except Yangtze River), Mississippi River, Yangtze River, Arctic, Australia (inland freshwaters), New Zealand (inland freshwaters), Indo-Pacific (Indian Ocean and the archipelago of Indonesia, Malaysia and Philippines), Africa (inland freshwaters), North America (inland freshwaters except the Laurentian Great Lakes, St Lawrence and Mississippi Rivers), South America (inland freshwaters), Ponto-Caspian region and unknown region.

To confirm species-specific information, we used the ISI Web of Science (WoS) platform by applying the following key synonymous terms: non-native OR alien OR exotic OR non-indigenous OR introduced OR colonizing OR invasive OR nonnative OR nonindigenous. Each species name was checked in combination with these terms, using AND as a combination type. Each publication was then exhaustively checked to determine habitat types, geographical origins and invaded regions attributable to each alien species. This literature search was performed only for species which were reported as alien, while habitat types from the World Amphipod Database were recorded in the case of all native species not recorded as alien. We excluded species for which no habitat or regional information was available, analysing salinity tolerances and origin regions of 884 and 880 species, respectively.

We statistically tested the null hypotheses that the proportions of gammarid species that are reported as alien and reported as non-alien are (i) equal among habitat types that they predominantly tolerate in their native range (freshwater, salt-tolerant (brackish), marine); and (ii) equal among geographical origins (Ponto-Caspian and ‘other'). We used contingency tables populated with raw frequency data with *χ*^2^-squared tests and assessed statistical significance at an *α* of 0.05.

Separately, a chord diagram was produced to illustrate flows of species among geographical regions. Here, as several alien species were native to or had invaded multiple regions, species numbers were divided among regions where appropriate, based on a per-species contribution of 1. For example, if a species was native to two regions, a value of 0.5 was attributed to each region; this was further subdivided if the same species invaded multiple regions. This ensured that a given species was not overrepresented graphically, eliminating potential biases among geographical regions.

## 3. Results

Total known gammarid diversity amounted to 918 species distributed across 25 families in aquatic environments (table 1). The richest families overall were Gammaridae, Acanthogammaridae and Eulimnogammaridae, with over 100 species each. Freshwater habitats were primarily tolerated by 82.8% of all gammarid species, brackish habitats by 6.3% and marine habitats by 10.9% (table 1), with alien species reported within five families (Gammarellidae, Gammaridae, Iphigenellidae, Micruropodidae and Pontogammaridae) (table 1). In total, 27 species were reported as being aliens, amounting to 2.9% of total known global gammarid diversity. The richest families for alien species were Gammaridae (4.6% of species in that family; 18 out of 391) and Pontogammaridae (13.9%; 5 out of 36).

**Table 1.** Global aquatic gammarid (Amphipoda: Gammaroidea) diversity across families, with total numbers of species and numbers of alien species. (Habitat type tolerances associated with all taxa within families (freshwater, salt-tolerant (i.e. freshwater up to brackish) and marine). Families with known reported alien species are emboldened.)

family	no. species	no. aliens	habitat (%)		
			freshwater	salt-tolerant	marine
Acanthogammaridae	123	—	99.2	0.8	0.0
Anisogammaridae	61	—	60.0	8.3	31.7
Baikalogammaridae	1	—	100.0	0.0	0.0
Bathyporeiidae	24	—	0.0	0.0	100.0
Behningiellidae	4	—	0.0	100.0	0.0
Carinogammaridae	1	—	100.0	0.0	0.0
Crypturopodidae	37	—	100.0	0.0	0.0
Eulimnogammaridae	113	—	100.0	0.0	0.0
Falklandellidae	3	—	100.0	0.0	0.0
Gammaracanthidae	4	—	50.0	50.0	0.0
**Gammarellidae**	**6**	**1**	**0.0**	**0.0**	**100.0**
**Gammaridae**	**391**	**18**	**77.7**	**10.3**	**12.0**
**Iphigenellidae**	**3**	**1**	**33.3**	**33.3**	**33.3**
Luciobliviidae	1	—	100.0	0.0	0.0
Macrohectopidae	1	—	100.0	0.0	0.0
Mesogammaridae	7	—	71.4	0.0	28.6
**Micruropodidae**	**43**	**2**	**100.0**	**0.0**	**0.0**
Ommatogammaridae	4	—	100.0	0.0	0.0
Pachyschesidae	16	—	100.0	0.0	0.0
Pallaseidae	21	—	95.2	4.8	0.0
Paraleptamphopidae	5	—	100.0	0.0	0.0
Phreatogammaridae	6	—	83.3	0.0	16.7
**Pontogammaridae**	**36**	**5**	**86.1**	**13.9**	**0.0**
Sensonatoridae	1	—	100.0	0.0	0.0
Typhlogammaridae	6	—	100.0	0.0	0.0
**total**	**918**	**27**	**82.8**	**6.3**	**10.9**

**Figure 1.**
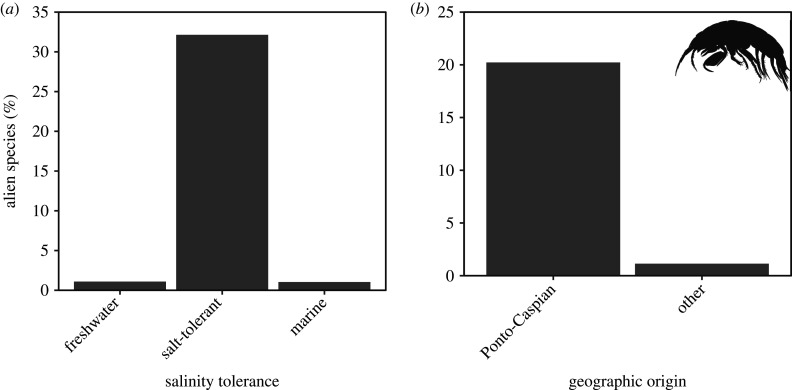
Bar plots illustrating (*a*) proportions of alien species within each originating habitat tolerance type, and (*b*) proportions of alien species from each geographical region. Note for (*a*) that ‘salt-tolerant' taxa withstand freshwater up to brackish water conditions, and ‘marine' taxa tolerate up to fully marine conditions. For (*b*), ‘other' regions include all biogeographic areas outside of the Ponto-Caspian region.

Significantly disproportionately more alien gammarids, relative to non-alien gammarids, originated from brackish waters (32.1%; 18 out of 56) compared to freshwater (1.1%; 8 out of 732) and marine environments (1.0%; 1 out of 96) (*χ*^2^ = 170.85, *p* < 0.001; figure 1*a*; 2 × 3 contingency table in the electronic supplementary material).

Similarly, significantly disproportionately more alien gammarids, relative to non-alien, originated from the Ponto-Caspian region (20.2%; 18 out of 89) compared to ‘other' regions (1.1%; 9 out of 791) (*χ*^2^ = 97.99, *p* < 0.001; figure 1*b*; 2 × 2 contingency table in the electronic supplementary material).

**Figure 2.**
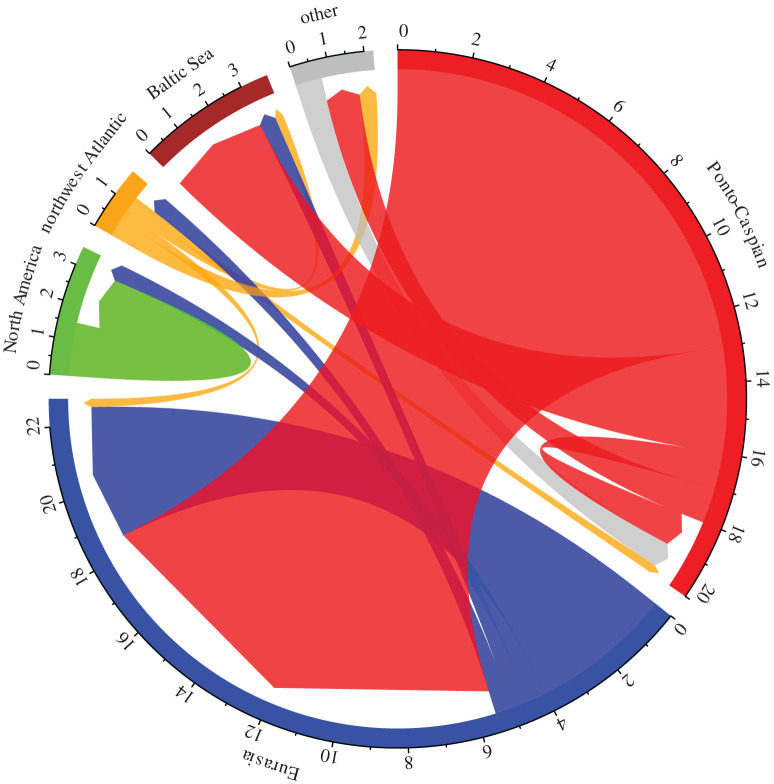
Alien gammarid species numbers flowing among different regions. The arrows at the end show the direction of each flow. Regions with no reported alien species are excluded. Eurasia and North America represent freshwater habitats. Flows within regions reflect invasions of distinct areas within those same regions. Single species which originated from, or invaded, multiple regions were divided among those regions to ensure equal contributions from each taxon. Note that the ‘other’ areas include the Mediterranean Sea, North Sea, northeast Atlantic and Great Lakes-St Lawrence River regions.

Flows of alien species from the Ponto-Caspian region (66.7% of all aliens; 18 out of 27) thus dominated (figure 2), exceeding flows from Eurasian freshwaters (20.4%; 5.5 out of 27), North American freshwaters (5.6%; 1.5 out of 27), northwest Atlantic (4.6%; 1.25 out of 27) and other habitats (2.8%; 0.75 out of 27). Major geographical regions, including African freshwaters, South American freshwaters and the entire Pacific, were the origin of no alien gammarids. Just one species, *G. angulosus*, invaded from the Mediterranean Sea, North Sea and northeast Atlantic regions, to the Ponto-Caspian region.

Eurasian freshwaters were most invaded overall (63.9% of alien species; 17.25 out of 27), and predominantly by Ponto-Caspian taxa (75.4% of that number; 13 out of 17.25), but also by other Eurasian species that were native to different areas within the region (23.2%; 4 out of 17.25) and Northwest Atlantic species (1.5%; 0.25 out of 17.25) (figure 2). By contrast, the Ponto-Caspian region was invaded by just 7% of known alien species globally (7.4%; 2 out of 27). The Baltic Sea was the second most frequently invaded system by alien gammarids (13.9%; 3.75 out of 27), followed by north American freshwaters (7.4%; 2 out of 27). Remaining ecosystems, including the Mediterranean Sea, North Sea and northwest Atlantic, had relatively few invasions (7.4% collectively; 2 out of 27); many others (see above list) contained no documented alien species.

## 4. Discussion

Alien gammarids are disproportionately represented by salt-tolerant (i.e. brackish) species, with freshwater and marine species proportionately rarely documented as being alien. Thus, fully freshwater and marine environments do not appear to be major ‘donor’ habitats for this taxonomic group, although the high number of freshwater species has resulted in some invaders from this pool of species. Moreover, profound biases were found regionally, with the vast majority of alien gammarids native to the Ponto-Caspian region, and high proportions invading Eurasian freshwaters, North American freshwaters and Baltic Sea waters. As such, we provide new lines of evidence that suggest environmental characteristics of specific donor regions of alien species are important predictors of invasion dynamics, with salt-tolerant Ponto-Caspian taxa particularly pervasive in their movement from brackish habitats to other brackish and freshwater locations [12,17]. Indeed, previous work has shown species interactions and impacts of gammarids can be driven by the influence of salinity on physiology and behaviour [21].

While gammarids have been important flagship species in studies of invasion success and invader impact, e.g. [19], this study found that only a relatively small proportion (3%) of the overall gammarid diversity is reported as alien. However, this alien species number is still relatively high when compared with certain aquatic species groups, such as insects [22]. Nevertheless, despite relatively high numbers of studies concerning these taxa (i.e. WoS search ‘Amphipoda' with above synonyms yields 355 studies (June 2020)), few species have successfully established outside of their native range. However, this could also indicate that many species have not yet had the opportunity to translocate and invade new environments [4]. Alien gammarids were mostly represented by the Gammaridae and Pontogammaridae families, which contain the majority of Ponto-Caspian taxa from this study. While it is possible that reduced recorder effort concerning other families influences known numbers of alien taxa, reported taxonomic biases here are stark and suggest certain families may have specific traits that promote aquatic invasion success, or are concentrated in localities that are highly interconnected [10,12]. Indeed, large shipping ports are often located in brackish water areas [12], potentially increasing invasion likelihoods to other habitats. This is despite 94% of gammarid diversity being associated with solely freshwater or marine environments.

The asynchronous movements of alien species suggest that fully freshwater and marine gammarids present a low invasion risk, while brackish species are particularly pervasive aliens. Numbers of brackish-origin alien species are particularly marked, given that just 6% of all gammarid species populate these environments, with 32% of salt-tolerant species known aliens. However, in the geographical context, 15 out of 18 alien gammarids of brackish origin originated from the Ponto-Caspian region, which has experienced highly changeable abiotic conditions owing to the complex geological history of the area [11,12]. Indeed, the diverse historical environmental regimes, which include freshwaters, that are experienced by taxa native to this region may provide an evolutionary predisposition to invade freshwater or brackish environments, as has occurred in Eurasian freshwaters, the Baltic Sea, as well as the Great Lakes-St Lawrence River [12,23]. This phenomenon has been further evidenced experimentally and by field observations, with Ponto-Caspian taxa highly tolerant to both freshwater and brackish habitats [17,18], and known to invade more systems than expected based on environmental matching and shipping frequency (i.e. independent of propagule pressure) [12]. Alternatively, while these species probably principally dispersed following human-mediated canalization and increased shipping intensity [23], Ponto-Caspian taxa are additionally known to have been introduced directly into Eurasian freshwaters in certain instances to promote fish farming [24]. Finally, recent genomic analyses suggest that crustaceans may readily evolve to fluctuating habitats thus promoting invasion success [25].

Comparatively few alien gammarids were found to have originated outside of the Ponto-Caspian, Eurasian, North American and northwest Atlantic regions. In turn, many entire geographical regions (e.g. Africa, Pacific Ocean and Southern Ocean) contributed, or received, zero reported alien gammarid species. These stark regional differences might be indicators of neglected areas for aquatic invasive species research more generally, or a lack of geographical connectivity. Moreover, with climate change projected to alter salinity regimes of waterbodies in future, our results suggest that those regions with projected desalinization trends will be most at risk of alien species e.g. [26]. Conversely, salinization of freshwaters is an emerging issue that could provide novel habitats for salt-tolerant taxa [27]. While this study found marked differences in alien species establishment dynamics according to habitat types and regional differences, future works should examine other species groups and habitats with known alien taxa to generalize findings across taxonomic groups. These efforts could help direct measures for mitigating the ongoing erosion of global biodiversity and other detrimental impacts caused by alien species.

## Supplementary Material

Supplementary Material 1

## Data accessibility

A data file can be found in the electronic supplementary material.
